# Trainee led intensive care unit induction - an educational and quality improvement project

**DOI:** 10.1186/2197-425X-3-S1-A865

**Published:** 2015-10-01

**Authors:** J Sumpter, JD Wijesuriya, R Mccomb, G Thomas-Black, A Achilleos, A Connell

**Affiliations:** Whittington Hospital, Anaesthetics & Intensive Care, London, United Kingdom; University College London Hospital, London, United Kingdom

## Introduction

Under the leadership of the Faculty of Intensive Care Medicine, junior trainees from varied medical backgrounds are increasingly undertaking placements in Intensive Care Units (ICU) [1]. Unlike their predecessors, current trainees may have little prior training in advanced organ support (AOS), yet clinical responsibility may be high - a potential patient safety issue. Traditional senior-doctor led departmental induction may not rapidly adapt to the evolving requirements of current trainees [2].

## Objectives

To assess the background, prior experience & confidence at managing AOS in a cohort of trainees at our institution. Satisfaction with the standard departmental induction programme (IP1) was also assessed.

To design a new induction programme (IP2); novel in the sole use of current unit trainees for its development & delivery, incorporating feedback from the initial assessment & tailored to the needs trainees from differing medical backgrounds.

To assess trainee confidence/satisfaction following IP2 & ensure project continuity.

## Methods

Three written assessments were completed by two cohorts of trainees; Cohort 1 was assessed post IP1, Cohort 2 was assessed pre & post IP2.

IP2 design was based on feedback from Cohort 1 & took a structured approach to AOS, with five system-based presentations & a written handbook. IP2 was delivered as a half-day of interactive lectures prior to Cohort 2 commencing placements in ICU.

Electronic copies of IP2 content were handed over to Cohort 2 to facilitate future delivery.

## Results

Cohort 1 (n = 8) included: 2× Core Medical trainees (CMT), 2× Emergency Medicine (EM) trainees & 3× foundation trainees (FT); with prior ICM experience: 3× none, 5× < 6 months. Cohort 2 (n = 6)) included: 1× CMT, 3 × FT & 2× anaesthetic trainees (AT); with prior ICM experience 3× none, 2× < 6 months & 1× < 12 months.

Assessment responses graded 0-5 (poor-excellent) were mean averaged. Pre & post IP2: trainee confidence at routine AOS management improved from 1.93 to 3.17; confidence at emergency AOS management improved from 2.17 to 3.33; understanding of AOS principles improved from 2.17 to 3.5. Overall satisfaction with induction improved from 2.38 to 4.83. (See Graph 1)

## Conclusions

As ICM training expands in the UK, junior trainee confidence & experience cannot be taken for granted. Induction programmes tailored for trainees of differing medical backgrounds can improve knowledge, confidence at managing AOS & improve trainee satisfaction.

Such programmes need not be costly or consultant delivered. Our trainee led & delivered programme resulted in improved levels of trainee confidence, high levels of satisfaction & provided a legacy of teaching opportunity for trainees within the unit.

**Figure 1:**
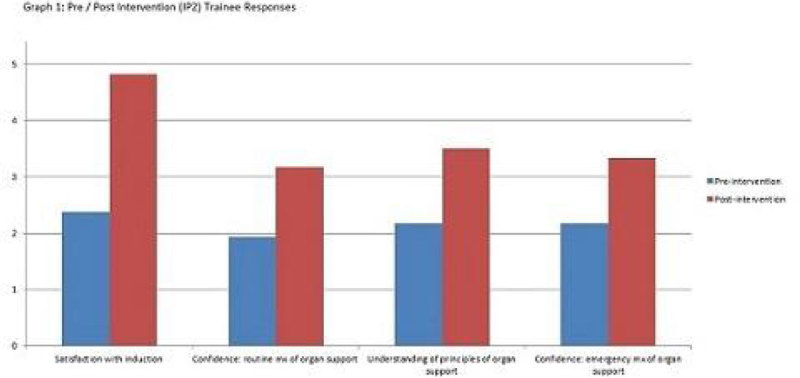
Figure 1
